# Inferring fruit infestation prevalence from a combination of pre-harvest monitoring and consignment sampling data

**DOI:** 10.1038/s41598-024-63569-9

**Published:** 2024-06-06

**Authors:** Peter Caley, Daniel W. Gladish, Lloyd Kingham, Rieks D. van Klinken

**Affiliations:** 1 CSIRO Data61, GPO Box 1700, Canberra, ACT 2601 Australia; 2 CSIRO Data61 GPO, GPO Box 2583, Brisbane, QLD 4001 Australia; 3 NSW Department of Primary Industries, Locked Bag 21, Orange, NSW 2800 Australia; 4 CSIRO Health & Biosecurity, GPO Box 2583, Brisbane, QLD 4001 Australia

**Keywords:** Statistics, Data integration

## Abstract

International trade in horticultural produce happens under phytosanitary inspection and production protocols. Fruit inspection typically involves the sampling and inspection of either 600-pieces or 2% of packed product within a single consignment destined for export, with the purpose of certification (typically with 95% confidence) that the true infestation level within the consignment in question doesn’t exceed a pre-specified design prevalence. Sampling of multiple consignments from multiple production blocks in conjunction with pre-harvest monitoring for pests can be used to provide additional inference on the prevalence of infested fruit within an overall production system subject to similar protocols. Here we develop a hierarchical Bayesian model that combines in-field monitoring data with consignment sample inspection data to infer the prevalence of infested fruit in a production system. The results illustrate how infestation prevalence is influenced by the number of consignments inspected, the detection efficacy of consignment sampling, and in-field monitoring effort and sensitivity. Uncertainty in inspection performance, monitoring methods, and exposure of fruit to pests is accommodated using statistical priors within a Bayesian modelling framework. We demonstrate that pre-harvest surveillance with a sufficient density of traps and moderate detection sensitivity can provide 95% belief that the prevalence of infestation is below $1 \times 10^{-6}$. In the absence of pre-harvest monitoring, it is still possible to gain high confidence in a very low prevalence of infestation ($<1 \times 10^{-5}$) on the basis of multiple clean samples if the inspection sensitivity during consignment sampling is high and sufficient consignments are inspected. Our work illustrates the cumulative power of in-field surveillance and consignment sampling to update estimates of infestation prevalence.

## Introduction

Inspection of consignments is a key component in the certification process to underpin trade in horticultural produce, where a consignment is defined as “A quantity of plants, plant products or other articles being moved from one country to another and covered, when required, by a single phytosanitary certificate.”^1^. Methodologies for the sampling of consignment lots are described in International Standards for Phytosanitary Measures (ISPM) No. 31 *Methodologies for sampling of consignments*^2^. A key objective of sampling of consignments is to “provide assurance that the number of regulated pests or infested units in a consignment does not exceed the specified tolerance level for the pest.”^2^. The choice of tolerance level (more generally called the “design prevalence”), which is the prevalence that one wishes to be able to detect with a given level of certainty (also expressed as statistical power), is central to this approach. Ultimately, the level of consignment sampling depends on the level of certainty desired. For example, for a large and well mixed consignment, sampling 600 items (i.e. individual fruit) with a test/inspection of perfect sensitivity (“Efficacy of detection” in ISPM 31 parlance) will detect an infestation prevalence of 0.5% with 95% confidence. This 600-piece sample from the consignment is a commonly required level of sampling^3^, and as practiced is essentially confirmatory: it provides a specified level of confidence that the consignment being sampled is not badly infested. It can be thought of as an exceedance threshold probability approach to risk management, and is a form of acceptance/compliance sampling^4^.

Under the ISPM 31 methodology, the specification of a design prevalence a priori simplifies the frequentist approach for making inference. A further attraction of the compliance sampling approach is its simplicity: valid inference only requires knowledge of a single parameter (the detection sensitivity $\delta$) along with the sole assumption that either the sample from the consignment is taken at random, or that the fruit within the consignment are “well” mixed.

In many situations for pests of quarantine concern, confidence in a much lower prevalence than the commonly chosen design prevalence of 0.5% is required, that along with the 600-piece sample size can be considered arbitrary^5^. Furthermore, there is interest from a broader risk analysis perspective in knowing what the most likely infestation level in a consignment (or a consignment pathway more generally) may be regardless of whether pests are detected or not^3,5^, the estimation of pest slippage rates^6^ and the associated implications for pest introduction risk^7^. To do this requires that key phytosanitary measures across the production cycle potentially impacting on the risk of infestation/infection of the produce are accounted for, such as in-field surveillance of pest populations. Such multiple measures are often part of a Phytosanitary Systems Approach—defined by the International Plant Protection Convention (IPPC) as being where two or more independent, risk-reducing management measures are combined to achieve the appropriate level of phytosanitary protection to achieve trade^8^. They are widely used to support trade in fresh horticultural produce^9,10^. Systems approaches can combine a diverse range of measures, but most include sampling of consignments^10^, most often in combination with in-field surveillance of pest populations.

In this paper we compare the inference on infestation prevalence arising from pre-harvest monitoring and compliance sampling alone and in combination. As inference can be weak at the level of a single consignment, we examined the value of broader inference arising from the sampling of multiple consignments and in-field pre-harvest monitoring (at different trap densities) from multiple blocks across a production system where the pest infestation levels in blocks within an orchard are related. The need to integrate over uncertainties in parameters and prior infestation prevalence naturally leads to a Bayesian approach to estimation, for which methods are increasingly well developed^11^. Here we develop a Bayesian model implemented in the R software environment^12^ for making inference on the prevalence of pest infestation. We restrict our inference to scenarios arising from well managed production systems where the number of detected infested pieces within a packed product and the number of pests detected within orchards pre-harvest is very low, with a focus on scenarios where no pests are detected via either monitoring method.

## Results

### Model development and evaluation

For the Bayesian model we developed, we assessed the adequacy of convergence of the MCMC chains for estimating key parameters of interest ($\pi$—prevalence of infestation; $\lambda$—propensity of pests to infest fruit) (see Table 1) across the range of scenarios using the convergence statistic of Gelman & Rubin^13^. Critically, as interest is in determining the resulting pest prevalence under “clean” in-field detection and consignment inspections, data are assumed to detect no pests in field and no infested fruit in consignment inspections in order to assess model convergence. That is, all observations are set to 0 for all blocks. Using 100,000 iterations for each MCMC chain following burn-in resulted in good convergence for the estimation of $\lambda$ across all $n=1296$ parameter combinations we assessed ($\bar{R}=1.0001$) with no values of *R* greater than 1.1. The degree of convergence for estimating $\pi$ we considered to be adequate ($\bar{R}=1.013$) with $97.5\%$ of *R* values less than 1.1 and $99.7\%$ less than 1.2.

Ideally we could test our model using real world data with known pest prevalences. However, to our knowledge data does not exist. Thus, to test our model’s ability to correctly infer the mean prevalence, we use simulated data. This is done by simulating from a realistic, yet known true pest prevalence, and potential resulting observations (in the form of pests detected per block and positive samples per consignment). Critically, this simulation process is realistic but notably a different formulation than our specified hierarchical Bayesian model. Data were generated using a range of Poisson intensities of pests per block (0.5–100) with the Poisson intensity for the mean number of fruit infested per pest Gamma-distributed (shape = 15, scale = 2). This differs from the Bayesian model we have specified for which the central tendency is less well known, and a Uniform distribution used for the mean intensity. That is, the distribution of pests per block was gamma-Poisson. This generated underlying true/actual prevalences (averaged over all blocks) in the range $2 \times 10^{-7}$ to $1 \times 10^{-4}$. The number of blocks was fixed at 10 with 10 consignment samples inspected per block, and five traps per block.

**Figure 1:**
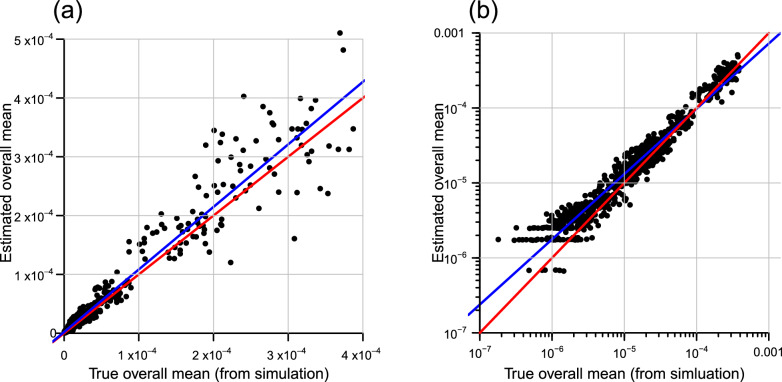
Relationship between estimated and known prevalence from simulated data on (**a**) linear, and (**b**) logarithmic scales. The red line in both plots is the line of equivalence with the blue line the linear model of best fit. Horizontal bands arise from the response variable for the sample data being able to take the same values (data is discrete) for a different underlying true prevalence.

**Figure 2:**
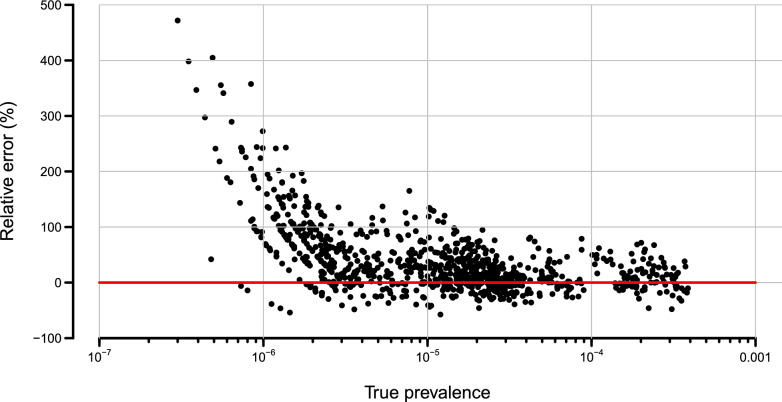
Relationship between the relative error between estimated and true underlying prevalence. Red horizontal line equates to zero bias. Note that bands arise from the sample data being able to take on the same values (data is discrete) for different underlying true prevalences.

As expected, the model was able to estimate the true prevalence over nearly four orders of magnitudes with adequate precision within the agricultural biosecurity context (Fig. 1a). There was, however, a positive bias that became more pronounced as prevalence became very low (Fig. 1b). The size of the relative error (i.e. $\frac{\hat{\pi }-\pi _{\text {True}}}{\pi _{\text {True}}}$) increased as the true prevalence drops below $1 \times 10^{-6}$, and the estimates became more positively biased (Fig. 2). Such bias would be expected given the uniform prior support on [0, 1] provided by the Beta(1,1) distribution. We return to the significance of this in the “Discussion”.

### Effect of in-field (pre-harvest) monitoring on inferred prevalence

**Figure 3:**
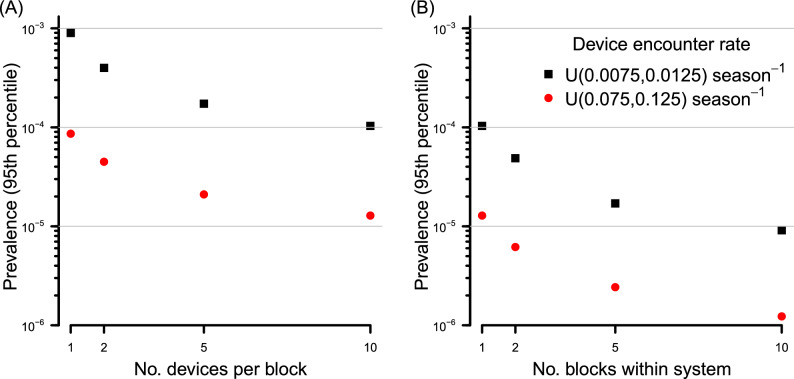
The effect on the inferred 95th prevalence percentile (assuming no pests are caught) for (**A**) A single block with an increasing density of traps to a maximum of 10 with either a low or high trap encounter rate, and (**B**) maintaining the density of traps per block at 10, though increasing the number of blocks within the production system. Calculations assume $10 \times 10^6$ fruit per block. There is no consignment sampling with associated inspection.

Unsurprisingly given the model structure, for a given set of pest abundance data (pests per production block), fecundity parameters (fruit infested per pest) and detection monitoring data (rate of encounter of pests with traps, traps per block, fruit inspection sensitivity), altering the density of fruit within a production system influences the inferred prevalence in an inverse linear manner due to the effect of dilution (results not shown). Similarly, the rate at which pests encounter traps and the density of surveillance traps per block influence estimated prevalence in a log-linear manner (Fig. 3A). Combining the effects of in-field (pre-harvest) pest surveillance across multiple related blocks can generate considerably stronger belief in a lower prevalence of infestation than for a single block, if the rate at which pests encounter and are detected by traps is high enough. For the upper range of the trap density and attractiveness of traps explored here, the inferred upper 95th percentile for the prevalence within the packed consignment approaches $1 \times 10^{-6}$ (Fig. 3B).

### Effect of multiple consignment sampling on inferred prevalence

The effect of fruit density on inference arising from the inspection of consignment samples alone was minimal (not shown). This arises from well known large sample effects coming in to play. Henceforth, we focus on production systems with the higher density of fruit ($10 \times 10^6$ per block). This is a density consistent with a cherry production system.

**Figure 4:**
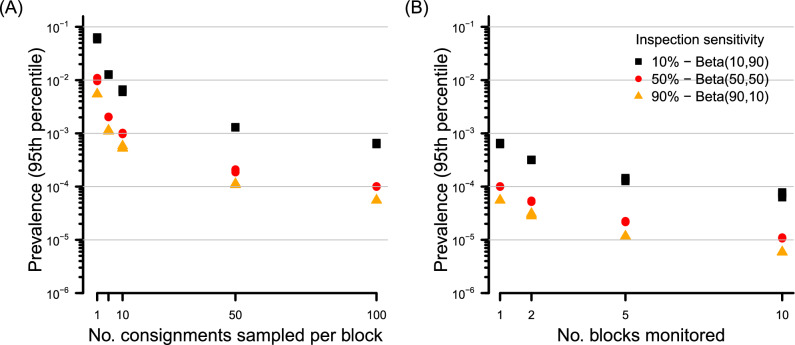
For three contrasting levels of consignment sampling inspection sensitivity, the effect on the inferred 95th percentile for the prevalence of infested fruit of (**A**) increasing the number of 600-piece consignment samples per block up to 100 per block, and (**B**) maintaining the number of 600-piece consignments per block at 100, and increasing the number of blocks within the production system. There are no in-field surveillance traps, $10 \times 10^6$ fruit per block and no pests are detected within any consignment sample.

As would be expected, both increasing the sensitivity of consignment sampling to detect infested fruit and the number of consignments sampled has a large effect on the inferred prevalence. The upper 95th prevalence percentile differed by an order of magnitude difference between a mean inspection detection sensitivity of 10% versus 90% (Fig. 4). Increasing the number of consignments sampled for which inspection returns clear results (in the absence of any in-field monitoring) reduces the inferred 95th prevalence percentile to as low as $c.~1 \times 10^{-4}$ if inspection sensitivity is high (90%), versus $c.~1 \times 10^{-3}$ for a low (10%) sensitivity consignment inspection (Fig. 4). Extending this increased number of consignments sampled per block (100) to a larger production system of 10 blocks (still in the absence of in-field monitoring) steadily lowers the inferred 95th percentile, to $c.~1 \times 10^{-4}$ for low sensitivity inspection, and $c.~1 \times 10^{-5}$ for high sensitivity consignment inspection (Fig. 4).

### Combined inference from in-field monitoring and consignment sampling

**Figure 5:**
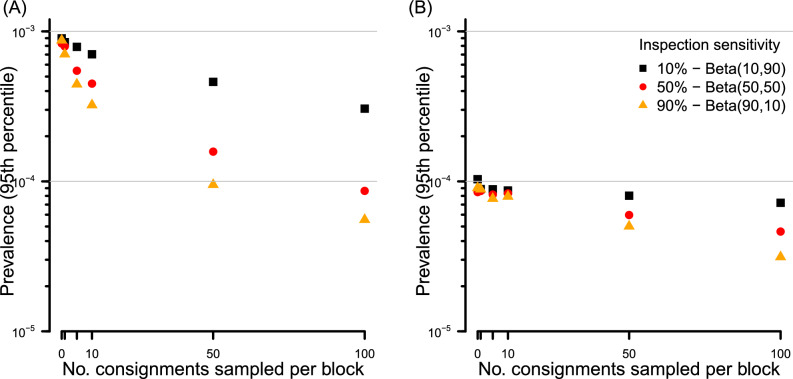
Effect of combining inference from both consignment sampling and pre-harvest monitoring in a production block containing (**A**) a single trap with $\tau \sim \mathscr {U}(0.0075,0.015)~\text {season}^{-1}$ and (**B**) ten traps with $\tau \sim \mathscr {U}(0.0075,0.015)~\text {season}^{-1}$. There are $10 \times 10^6$ fruit per block, and no detections in either in-field traps or consignment samples.

**Figure 6:**
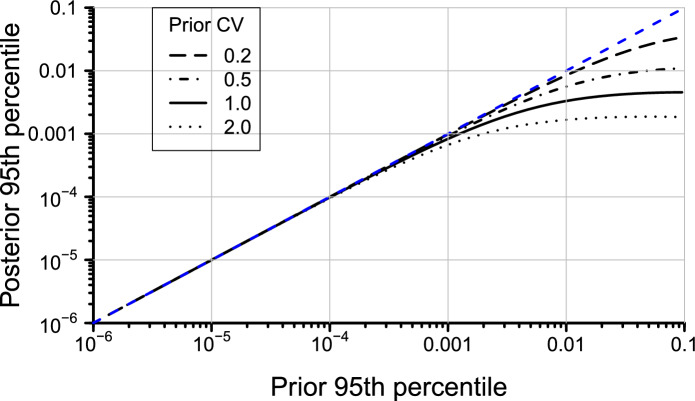
Posterior prevalence in relation to prior prevalence (with varying levels of relative precision (CV) held constant across the range of *p* in each instance) following a negative (i.e. clear) inspection of a sample of 600 fruit selected randomly, assuming 100% efficacy of detection. Blue dashed line is line of equivalence between the prior and posterior. Note the logarithmic axes.

For pests that have a reasonable encounter rate with traps (e.g. 0.01 season^-1^), we have already shown that the deployment of in-field surveillance pushes the inferred prevalence to *c.* $1 \times 10^{-4}$ or lower at high fruit density (Fig. 3). At this level of prior belief, our previous finding (see Fig. 6) shows that a single consignment sample of typical size, even of high inspection sensitivity, will not modify the posterior prevalence by an appreciable amount. Of course, undertaking additional consignment inspection data, and extending the inference to multiple blocks using our model can further improve inference. It is where in-field monitoring is of low sensitivity that improvements are most marked, and directly related to the inspection sensitivity. For example, where a single trap of moderate attractiveness is deployed, combining the inference from multiple consignments sampled generates a meaningful reduction in the inferred prevalence (Fig. 5A). Such improvement is less marked where both the density of in-field traps is high along with their attractiveness to pests (Fig. 5B).

Where in-field surveillance intensity is high, and no pests have been detected across multiple blocks (e.g. Fig. 3), the additional benefits of consignment sampling is trivial (plot not shown). We note that our calculations use large-sample approximations, which is typically the case in horticulture. If, however, the size of consignments are small, then finite-size corrections may become relevant.

## Discussion

In this study, we developed a model that incorporated pre-harvest monitoring and the inspection of post-harvest consignment sampling conducted over multiple blocks and using spatial random effects. In doing so, we have shown how combining data from multiple sources can be used to make quantitative inference on the prevalence of infested fruit. This is an important extension from the current compliance-based sampling approach that typically generates inference for a single consignment only. The work has direct application to systems approaches, which typically include pre-harvest monitoring with post-harvest inspection measures^10^, though quantitative methods to incorporate these measures in an inferential framework is lacking. Our analyses show that combining the data from multiple blocks and consignments can provide strong evidence for the absence or very low infestation prevalence of pest species that can be detected pre-harvest using in-field devices such as traps, or their infested fruit are detectable during consignment sampling. We note that the relative bias of our estimated prevalence using our model increases as the true prevalence become very low—an effect of the prior on the prevalence. From an agricultural biosecurity risk analysis perspective this estimation bias would be considered conservative (i.e. acceptable).

Obtaining the estimated posterior distribution for the prevalence of infestation within a consignment has additional uses from a risk management perspective beyond simply knowing the probability that an arbitrary design prevalence has been exceeded (though note that this can be calculated directly from the posterior). An obvious use for estimates of the posterior distribution is to determine how efficacious subsequent post-harvest treatments (e.g. cold treatment or fumigation) would need to be to reduce the prevalence below an acceptable level.

Although we have presented inference arising from pre-harvest monitoring and post-harvest consignment sampling as if they are potentially equivalent, the utility of the inference they provide differs. Pre-harvest monitoring results across multiple blocks are known at the time of harvest (or shortly thereafter) and hence can be used in the prospective sense. In contrast, consignment sampling inference arising from multiple samples and blocks are not all available until the packing process is completed (and the consignments sent). Such knowledge, however, can be used subsequently as a prior, with the main assumption in relation to inference being that the conditions in the current year are similar to those of the previous year. This is essentially an empirical prior, and indeed, this could be used as a prior in the model we have presented here. Updating the prior distribution based on previous seasons harvest may not make sense for some cases, however. Alternatively, and perhaps more fittingly, inference could be updated (a “rolling” estimate) as consignments are packed and samples are inspected.

The upper limit for the density of traps per block we have explored here (10 block^-1^) is higher, for example, than historical numbers used for fruit fly monitoring. However, with the development of remotely monitored electronic monitoring devices such as the McPhail-type “e-traps”^14^ or the RapidAIM sensor approach^15^, a much higher density of detection devices is now feasible, along with much more timely reporting. Our analysis illustrates how such devices can be deployed at higher densities to achieve high levels of confidence in a very low prevalence of infestation within a packed product.

Increasing the density of in-field detection traps (pre-harvest pest monitoring) within reasonable limits generates larger decreases in inferred prevalence than increasing the sensitivity of consignment inspections. Without in-field pest surveillance, 600-piece consignment sampling of low inspection sensitivity struggles to reduce the inferred 95th prevalence percentile to levels below 1 in 10,000, even after a considerable number of consignments (i.e. 100) are sampled for a number of blocks (e.g. 10). A high inspection sensitivity (e.g. 90%) reduces this by an order of magnitude, though we note that inspection sensitivity is rarely quantified^16^. It follows that if one was to extend the approach to incorporate pre-harvest inspection of fruit (e.g. as undertaken during crop scouting), there would be similarly low power to infer a low prevalence if the sampling fraction and inspection sensitivity are both low, unless sampling can be targeted towards vulnerable fruit. Accounting for latter would require quantification of the selectivity of targeted sampling beyond the scope of this paper. Consignment sampling, however, will remain important for pests that are hard to detect pre-harvest, and as a form of compliance checking. It is also worth noting that as packhouses become increasing automated, the ability for all (c.f. a sample) of fruit to be inspected is becoming a reality, and analyses are needed to accommodate for this.

The areal rate ($\tau$) at which pests encounter and are detected by in-field traps, along with the number of fruit infested per pest are key model parameters. Furthermore, the purpose of the model presented is not to estimate such parameters if they are largely unknown. They are indeed estimable if the number of pests within each block ($P_i$) is well known, but this is the latent variable we are most interested in estimating from a practical application viewpoint, and is the hardest to measure. The values for $\tau$ we have chosen are illustrative, ranging from what translates to a low of *c.* 1% capture probability per season to near certain capture at a trap density of 1 per unit area. For a specific pest, $\tau$ would take a prior distribution reflecting what is known about the sensitivity of the in-field surveillance device for detecting the pest in question. This can be calculated using observed capture probabilities from trapping devices laid out at a known density. For example, Table 3 in Fletcher^17^ documents the daily catch proportion of Queensland fruit fly (*Bactrocera tryoni*) at a known trap density of *c.* $6~\text {km}^{-2}$. A mean daily capture probability of 0.013 was observed, from which one can estimate $\tau \approx 0.0016~\text {km}^2~\text {day}^{-1}$ (see “Methods”). Uncertainty surrounding $\tau$ would ideally be represented using a Gamma distribution if there is informative prior information on the mean, or a uniform distribution as we have illustrated where less information about the mean is known though it is reasonable to put bounds on it. We note also that the results are conditional on the assumption that the movement of pests in relation to the spatial intensity of devices is such that all pests have a chance of encountering a device, regardless of how low the rate of encounter is. For specific pests and in-field surveillance devices, the time-based probability of capture and hence encounter rate (including uncertainty) can be calculated using the simulation modelling approach of van Klinken et al.^18^.

Our model can also be used to provide an estimate of the expected number of infested fruit within non-rejected consignments—termed “expected slippage” by Chen et al.^6^. Such information can feed directly into import risk assessments, where for internal pests in particular, there would be interest in inferring the distribution of the number of surviving larvae/eggs per fruit (or those able to complete development) as this influences the risk of successful pest establishment^7,19^.

In summary, we have illustrated a statistical model for quantitatively inferring the phytosanitary state of fruit produce sourced from a production system characterised by an initial pest infestation status, pre-harvest pest surveillance data and post-harvest consignment sampling inspection data. The model can be applied to multiple consignments sourced from multiple related blocks. The results illustrate the degree to which the application of these measures, either singularly or in combination, can provide confidence in the phytosanitary state of a fruit produce, depending on the relevant parameter settings. A natural extension of the model would be the inclusion of data on the climatic suitability at the production site for the pest species, which could be naturally incorporated by using an informed prior for the number of pests (*P*) rather than the current “flat” prior on the prevalence of infestation within fruit. The inference methods we have developed and presented here can be extended to account for the impact of other measures applied as part of a systems approach such as optical grading, and other treatments that are not considered sufficiently efficacious as a single measure^20^.

## Methods

### Production system description

#### Monitoring and sampling

We modelled a standardised representation of a horticultural production system at the level of farm enterprise (orchard), consisting of multiple production blocks. Pre-harvest (in-field) surveillance can occur within blocks from differing densities of traps. Following harvest and packhouse sorting/grading, multiple consignments (“packed product”) arise from within each block from which consignment samples can be taken for inspection. The sample size for sampling of consignments was set at the commonly chosen 600^3^, though note that this can be varied as desired. We further assumed, as with the calculations underpinning ISPM 31^2^ that sampling within a consignment is either undertaken randomly, or that the consignment is well mixed prior to sampling. This modelling resolution aligns with block-based certification and compliance.

Data used in our model are generated in two ways. First, data are generated in the operational context in which we are seeking to apply the model and make inference, particularly where there are no pests found during the inspection of samples taken from consignments and during pre-harvest monitoring. Inference to date has focused on a single sample (typically of size 600), but we extend this by generating datasets that include the inspections of multiple consignment samples arising from multiple production blocks — still with no pests found during any consignment sample inspection. Second, to test the ability of the model to make sound inference, we generate realistic datasets with a known (though stochastic) number of pests, and known (though again stochastic) parameters relating to the infestation of fruit. For these data, pests can be detected during pre-harvest monitoring and the inspection of consignments sampled.

#### The status quo—inference on infestation status from a standard 600-piece consignment sample

Before describing a statistical model to integrate additional information from in-field surveillance and related production blocks, we explore how the prevalence is updated based on a single 600-piece consignment sample with perfect inspection sensitivity where no infested fruit are found: the status quo. Posterior prevalences were calculated using the result that, when modelling the number of positive detections ($y=0,1,2,\ldots$) from a binomial distribution with sample size *n* from and $p \sim \text {Beta}(\alpha ,~\beta )$, the posterior distribution for *p* is distributed as Beta($\alpha +y$, $n-y+\beta$)^21^. Here we assume no detections (i.e. $y=0$), with the mean of the prior distribution for *p* ranging from $1 \times 10^{-6}$ to 0.1 with parameters chosen to ensure a constant coefficient of variation (CV ) equal to $\sqrt{\text {var}(p)}/E(p)$ across all values of *p* where:

$$\begin{aligned} \hat{\alpha }= & {} \frac{1-p}{CV^2} - p, ~~ \text {and} \\ \hat{\beta }= & {} \frac{(1-p)\hat{\alpha }}{p}. \end{aligned}$$

The results clearly illustrate that inspecting a small consignment sample from a large population cannot provide strong inference (belief) in a very low prevalence of infestation. For example, even with 100% detection sensitivity, inspecting a 600-piece sample does not substantially alter the prior belief in infestation prevalence, unless the believed infestation prevalence is high to begin with though imprecise (Fig. 6). As the prior prevalence decreases, the proportional change in belief arising from the clean sample diminishes. Indeed, once the upper 95% quantile for the prior prevalence drops below one in one thousand ($p=0.001$), the inferred reduction in prevalence arising from a negative (i.e. clear) 600-piece sample is negligible, even in situations where the precision on the prior value for *p* is low (Fig. 6). Inspection of a single 600-piece consignment sample thus serves as “confirmatory” measure that pest prevalence is unacceptably high.

### Statistical model description

#### General assumptions

It is assumed that inference is being made on a system where there is reasonable prior knowledge of both the biology of the pest in question, and sensitivity of the surveillance methods to either detect pests within the orchard during pre-harvest monitoring, or infested fruit during the inspection of samples from packed product (consignments).

#### Fruit infestation prevalence

The number of fruit infested (e.g. oviposited) per pest in block *i*, denoted $O_{i}$, is Poisson with intensity parameter $\lambda$:

$$\begin{aligned} O_{i} \sim \text {Pois}(\lambda ), \end{aligned}$$

where the prior for the intensity is:

$$\begin{aligned} \lambda \sim \mathscr {U}(\lambda _L,\lambda _U). \end{aligned}$$

The overall prevalence of infested fruit, denoted $\pi$, lies between 0 and 1 and thus assumed a beta distribution prior:

$$\begin{aligned} \pi \sim \text {Beta}(p_{a}, p_{b}). \end{aligned}$$

Within each orchard, we assume that the pest infestation levels within blocks are related as they are effectively part of a cluster, and that this relationship can be codified using a random effect, that we specify as normally distributed on the logit scale with a mean of zero and variance $\sigma ^2$. At the block level, prevalence is thus:

$$\begin{aligned} \text {logit}(\pi _i)= & {} \text {logit}(\pi ) + V, ~~\text {where} \\ V\sim & {} \mathscr {N}(0,\sigma ^2). \end{aligned}$$

Under this formulation, the hyperparameters $\lambda _L$, $\lambda _U$, $p_{a}$, and $p_{b}$ are user-specified. We note that when choosing prior distributions for random effects on a logit scale to model variation in a proportion, reasonably uninformative or “vague” $\mathscr {N}(0,\sigma ^2)$ priors may result in a strongly bimodel implicit prior value on $\pi _i$ for seemingly innocuous (i.e. large and hence “flat”) values for $\sigma$^22^. Instead, we place a standard uniform prior on the precision. That is:

$$\begin{aligned} \frac{1}{\sigma } \sim \mathscr {U}(0,1). \end{aligned}$$

**In-field surveillance:** Surveillance is undertaken and evaluated at the block level, with the sensitivity to detect pests (if present) determined by the number of pests ($P_{i}$) in block *i*, the density of traps within the block ($D_{i}$), and the effective rate of areal search ($\tau$) at which pests within the block may encounter (and be caught/detected by) traps over the period that fruit are susceptible to infestation. Over this period of fruit vulnerability, the expected rate of a pest encountering a trap and being captured is the product of $P_{i}$ and the probability of individual capture, defined as $p(c_{i}) \equiv 1 -\exp {(-\tau D_{i})}$. Note that if both $p(c_{i})$ and *D* are known or have been estimated, this relationship can be rearranged to estimate $\tau$ as:

$$\begin{aligned} \hat{\tau } = \frac{-\text {log}(1-p(c_i))}{D_i}. \end{aligned}$$

Such and estimate for $\tau$ could then be entered into the model as a prior distribution. For an individual block *i* within the period of pest vulnerability, the number of pests caught is modelled as Poisson:

$$\begin{aligned} X_i \sim \text {Pois}(P_{i} p(c_{i})), \end{aligned}$$

where the rate at which pests encounter traps is known within uniformly-distributed bounds:

$$\begin{aligned} \tau \sim \mathscr {U}(\tau _L,\tau _U). \end{aligned}$$

The number of infested fruit in a block ($I_i$) and the corresponding number of pests ($P_i$) are calculated as the derived quantities:

$$\begin{aligned} I_{i}= & {} \pi _{i} N,~~\text {and} \\ P_{i}= & {} I_{i} / O_{i}, \end{aligned}$$

where *N* is the number of fruit per block (assumed constant). For our purposes, though we do explore various values of *N*, we set *N* at $10 \times 10^6$ that is indicative of cherry orchards.

Note that our calculations assume that a trap (or detection device) may capture or detect an individual pest trapped once and once only. Should the model be generalised to detection devices that could detect the same pest individual multiple times (though not with unique identification), then the Poisson rate parameter for $X_i$ in the above would then be $P_i \tau D_i$ (c.f. $P_i p(c_i)$).

**Consignment-sampling:**

The number of infested units (*Y*) detected within *C* consignment samples of size *n* with inspection detection sensitivity $\delta$ can be well approximated using a Poisson distribution with rate parameter $\mu =\delta n C \pi$. This uses the well-known Poisson approximation to a Binomial for when *nC* is large relative to $\delta \pi$^23^. Note this approximation is used by Yamamura et al^24^ for modelling sampling inspection, and is considered conservative^25^. That is:

$$\begin{aligned} Y_{i} \sim \text {Pois}(\mu _{i}). \end{aligned}$$

The sensitivity of sampling will clearly depend on the type of fruit, pest (internal vs. external), stage of pest development, and inspection method (e.g. visual vs. destructive). There are little data on inspection sensitivity for most commodities and pests, and what data there are suggest that the detection sensitivity can vary widely^16^. Hence we explored three detection sensitivities within our scenarios—a low ($\bar{\delta }=$10%) represented by a Beta(10,90) distribution, a moderate ($\bar{\delta }=$50%) represented by a Beta(50,50) distribution, and a high ($\bar{\delta }=$90%) represented by a Beta(90,10) distribution. Ideally, trial data should be used to inform the parameterisation of the Beta distribution describing the detection sensitivity of fruit inspection.

A summary of model variables and parameters, their description, priors and rationale (if applicable) is provided in Table 1. The illustration of multiple choices for priors reflects the fact that different production systems will have different amounts of associated prior knowledge. We note that the form of priors can be flexible. For example, if the mean for the number of fruit infested per pest ($\lambda$) could be characterised using a Gamma distribution this could be easily accommodated with minor code modification. The values chosen for consignment inspection sensitivity ($\delta$) reflect realistic variation associated with pest type, commodity and inspection method.

We chose to place a prior on the prevalence of infested fruit ($\pi$), with no prior on the number of pests per block (*P*) for two reasons: one technical and the other for reasons of real-world acceptance. On the technical side, in practise $\pi$ and *P* will be strongly dependent (with the exact relationship mediated through the rate of infestation *O* but also the density of fruit), making the specification of appropriately correlated priors challenging. In regard to maximising the acceptance by intended end-users, in the general absence of ancillary data that could be used to support an informed prior for the number of pests within the orchard, choosing a uniform/vague prior for $\pi$ will be most easily accepted by end-users.

**Table 1:** Summary of model variables and parameters, their description, the values/priors taken, and the rationale for the choice.

Parameter/variable	Description	Value(s)/Prior*	Rationale
*B*	Number of production blocks per orchard	1–10	Illustrative range
*N*	Number of fruit per block^†^	$10 \times 10^6$	Illustrative of cherries
*C*	Number of sampled consignments per block	1–100	Typical range
*n*	Consignment sample size	600 (fixed)	Industry standard
$\delta$	Consignment inspection sensitivity (per individual piece of fruit)	Beta(10,90) Beta(50,50) Beta(90,10)	Low and informative Medium and informative High and informative
*P*	Number of pests per block	n/a	Derived
*O*	Number of fruit infested per pest	Pois($\lambda$) $\lambda \sim \mathscr {U}(20,30)$	Illustrative range for a serious pest that generates considerable *per capita* infestation
*I*	The number of infested fruit per block	n/a	Of primary interest for estimation, along with $\pi$
*D*	Density of traps within block	0–10 per unit area	Illustrative range
$\tau$	Effective area over which pests potentially encounter traps per season	$\mathscr {U}(0.0075,0.0125)$ $\mathscr {U}(0.075,0.125)$	Illustrative ranges for “low” and “high”
$\pi$	Overall prevalence of infested fruit	Beta(1,1)	Flat/vague prior — arguably acceptable to regulators
*V*	Block random effect for $\pi _i$ on logit scale	$\mathscr {N}(0,\sigma ^2)$ $1/\sigma \sim \mathscr {U}(0,1)$	Flat implicit prior support on [0, 1] for $\pi$ (see text)

*Distribution notation: $\mathscr {U}$—uniform, $\mathscr {N}$—normal.

^†^Calculated as the product of the density of fruit per unit area and the mean area per block. See R code within Supplementary Material.

#### Model fitting

Models were fitted using the “rjags” library^26^ for the software R version 4.0.2^12^. The full posterior to be sampled is:

1 $$\begin{aligned}{}[\pi ,O,\lambda , \delta | x,y,D,\tau ]\propto & {} [x |\tau , D] \nonumber \\\times & {} [y|\delta , \pi , C, n] \nonumber \\\times & {} [\pi |O, P, N] \nonumber \\\times & {} [O| \lambda ] \nonumber \\\times & {} [\delta ][\lambda ][\pi ][\tau ] \end{aligned}$$

where *x* is the in-field captures of pests and *y* the detections of infested fruit within sample from packed consignment.

It is improbable that all the parameters are jointly identifiable, and neither need they be, as it is assumed that it is possible to place informed empirical prior distributions on the fruit inspection sensitivity ($\delta$)^16^, pest fecundity ($\lambda$)^27^, and the rate ($\tau$) at which pests interact with traps^28^.

The R code for fitting the jags model is provided in the Supplementary Material.

#### Estimating fruit infestation prevalence

The mean and 95th percentile for the prevalence of infested fruit produced within the production system was estimated from the posterior distribution over all blocks (if more than one block involved).

### Supplementary Information

Supplementary Information.

## Data Availability

The datasets used and/or analysed during the current study are available from the corresponding author on reasonable request. No plant material was used in this study.
